# Evaluation of treatment response in extrapulmonary tuberculosis in a low-resource setting

**DOI:** 10.1186/s12879-019-4034-z

**Published:** 2019-05-16

**Authors:** Melissa Davidsen Jørstad, Anne Ma Dyrhol-Riise, Jörg Aßmus, Msafiri Marijani, Lisbet Sviland, Tehmina Mustafa

**Affiliations:** 1 Centre for International Health, Department of Global Public Health and Primary Care, University of Bergen, P.O. box 7804, N-5020 Bergen, Norway; 2 Department of Thoracic medicine, Haukeland University Hospital, Jonas Lies vei 65, N-5021 Bergen, Norway; 3 Department of Clinical Science, Faculty of Medicine, University of Bergen, Bergen, Norway; 4 Department of Infectious Diseases, Oslo University Hospital, Oslo, Norway; 5 Institute of Clinical Medicine, Faculty of Medicine, University of Oslo, Oslo, Norway; 6 Centre for Clinical Research, Haukeland University Hospital, Bergen, Norway; 7 Department of Diagnostic Services, Mnazi Mmoja Referral Hospital, Zanzibar, United Republic of Tanzania; 8 Department of Clinical Medicine, Faculty of Medicine, University of Bergen, Bergen, Norway; 9 Department of Pathology, Haukeland University Hospital, Bergen, Norway

**Keywords:** Treatment outcome, Clinical parameters, Weight gain, Tuberculous lymphadenitis, Tuberculous pleuritis

## Abstract

**Background:**

Diagnosing extrapulmonary tuberculosis (EPTB) is challenging and many patients are initiated on empirical anti-TB treatment without a laboratory confirmed diagnosis. Monitoring treatment response is thus important to ensure correct diagnosis and proper disease management. The definition of satisfactory response to treatment in EPTB remains unclear. The objectives of this study were to describe the clinical presentation of EPTB and the effect of treatment on clinical parameters. Further, to assess if simple clinical parameters, without laboratory data, could evaluate treatment response.

**Methods:**

Prospective cohort study of presumptive EPTB patients at Mnazi Mmoja Hospital, Zanzibar. By using a composite reference standard, patients were categorized as TB or non-TB cases. The TB patients were followed during anti-TB treatment.

**Results:**

There were 64 TB and 62 non-TB cases. The frequency of symptoms at baseline were comparable in TB and non-TB patients, with lymphadenitis and pleuritis as the most common manifestations. Among TB cases, there was a trend towards regression of lymphadenopathy after 2 months, and at treatment completion 24/28 (86%) cases showed full regression. Weight gain ≥5% was reported in 36/49 (73%) of the TB patients at 2 months and in 38/46 (83%) at treatment completion. After 2 months of treatment, a combination of clinical parameters; improvement of symptoms (50/50), ≥5% weight gain (36/49) and regression of physical signs (45/49) correlated with the treatment response.

**Conclusions:**

An algorithm including only simple clinical parameters could be used as an easy tool to assess treatment responses in low-resource settings. However, this needs to be tested on a larger sample size.

## Background

Globally, extrapulmonary tuberculosis (EPTB) accounts for 15% of the notified incident tuberculosis (TB) cases [1]. Laboratory confirmation of EPTB is challenging due to the paucibacillary nature of disease and limited resources in high-TB endemic settings. The diagnosis is therefore often made only on medical history and clinical signs and many patients are started on empirical anti-TB treatment. It is thus important to monitor response to treatment to ensure diagnosis and appropriate disease management. Monitoring response to treatment in EPTB is challenging and clinical assessment is generally recommended [2]. However, the definition of satisfactory clinical response to treatment among the various forms of EPTB remains unclear. Earlier studies have suggested a combination of clinical and laboratory criteria for evaluating response to treatment [3, 4]. Repeated invasive procedures or measurement of inflammatory markers are often incorporated in the definition of treatment response [4]. In low-resource settings with limited capacity for laboratory investigations and invasive procedures, it would be useful to assess response to treatment in EPTB by using simple clinical criteria.

In Zanzibar the estimated prevalence of bacteriologically confirmed pulmonary TB (PTB) among the adult population is 124/100000 [5]. In 2015, EPTB accounted for 24% of the notified TB cases [6]. In contrast to PTB, the EPTB patients are not followed systematically by the TB programme during anti-TB treatment. Symptoms and signs are often not documented in the TB records and the only information available is “treatment completed”, which does not always correspond to successful remission.

The objectives of this study were to describe the clinical presentation of EPTB among patients presenting at Mnazi Mmoja Hospital (MMH), Zanzibar, and to describe the effect of anti-TB treatment on clinical parameters. Further, to assess if simple clinical parameters, without laboratory support, could be used to reliably evaluate treatment response.

## Methods

### Study setting and population

This prospective cohort study was nested in a larger project at MMH [7], the main referral hospital in Zanzibar. Children and adults presenting with symptoms suggestive of EPTB were enrolled from in- and outpatient departments for a period of thirteen months starting 1st of August 2014. Patients were excluded if a specimen from the presumptive site of infection was not sent for laboratory investigations, if they did not give consent or had received anti-TB treatment during the previous year.

### Data collection

A medical history was obtained from the patients using their local language, Swahili, and a physical examination was performed at the time of inclusion and at follow-up visits after the intensive phase of anti-TB treatment at 2 months (median 71 days, 5–95 percentile 55–109 days) and at the end of treatment (median 175 days, 5–95 percentile 145–244 days). At inclusion, a pretested semi-structured questionnaire was used [7]. At the follow-up visits, clinical assessment was done without a standard questionnaire. Improvement of symptoms; cough, fever, appetite, fatigue, local symptoms and general condition reported by the patient and assessed by clinician, and results from the physical examination were registered in the patient’s study folder. Patients who were not started on anti-TB therapy were followed until a diagnosis was established or until recovery.

### Clinical investigation

Enlarged lymph nodes were measured by the size of the long axis of the largest swelling using a measuring tape, if not available, an eye estimate was used. Any change during anti-TB treatment in the size of nodes, appearance of new nodes, formation of fistulae and/or abscesses were recorded. Size of the lymph nodes was designated to one of four categories; not palpable (palpable lymph nodes < 1 cm was also included in this group), ≤2 cm, > 2–4 cm or > 4 cm. Residual lymphadenopathy after treatment was defined as palpable lymph nodes > 1 cm. Pleural or peritoneal effusions were evaluated clinically and by X-ray and ultrasound when available. All patients were weighed, and weight change was recorded in kilograms (kg) and as percentage change in weight at two intervals; 1) between treatment onset and 2 months of treatment, and 2) between treatment onset and end of treatment.

### Laboratory investigations

Acid-fast bacilli (AFB) microscopy, *Mycobacterium tuberculosis* (Mtb) culture, GeneXpert® MTB/RIF (Xpert) assay, and cytological/histological evaluation were performed on extrapulmonary specimens. In addition, other laboratory investigations such as bacteriological culture and Gram staining, and protein quantification and white cell count in effusions, were performed if available. CD4 cell counts and human immunodeficiency virus (HIV) ribonucleic acid levels were not available for the HIV positive patients.

### Composite reference standard for categorization of patients

A composite reference standard (CRS) was used to categorize the study participants as “confirmed TB cases” (positive Mtb culture and/or Mtb detected by the Xpert assay), “probable TB cases” (clinical presumptive EPTB and either response to anti-TB treatment or bacteriologically confirmed PTB, and one of the following; AFB on Ziehl-Neelsen stain of extrapulmonary specimen or cytology/histology consistent with TB or lymphocytosis on fluid cytology or radiological findings suggestive of EPTB), “possible TB cases” (clinical presumptive EPTB and one of the following; AFB on Ziehl-Neelsen stain of extrapulmonary specimen or cytology/histology consistent with TB or lymphocytosis on fluid cytology or radiological findings suggestive of EPTB or response to anti-TB treatment) and “non-TB cases” (negative Mtb culture and/or Mtb not detected by the Xpert assay and one of the following; improvement without anti-TB treatment or cytology/histology concluded other diagnosis than TB or alternative diagnosis concluded by the local clinician or non-response to anti-TB treatment) [7]. Patients unable to be categorized by the CRS were termed “uncategorized” and excluded from further analyses [7].

### Statistical analysis

Chi-square test was used in the analysis of categorical variables and Mann-Whitney U test and Kruskal-Wallis test for group-wise comparison of continuous variables. The general significance level was set to 0.05. Bonferroni correction was used to adjust for multiple testing effects leading to a marginal level of 0.0071 for symptoms (7 tests), 0.01 for presumptive TB lymphadenitis (5 tests) and 0.017 for weight gain (3 tests). Data were analysed using IBM SPSS Statistics for Windows, version 24 (Armonk, NY, USA), and graphics were created using Matlab 9.0 (Natick, MA, USA).

## Results

### Study participants

A total of 132 patients were enrolled and based on the CRS categorized as TB (confirmed, *n* = 12; probable, *n* = 34; possible, *n* = 18) and non-TB (*n* = 62) cases, while 6 patients were uncategorized and excluded from further analyses. Baseline sociodemographic and clinical characteristics are presented in Table 1.

**Table 1:** Characteristics of the study participants

	Total	Lymphadenitis		Pleuritis		Other sites of infection^a^	
	*n* = 126	*n* = 67		*n* = 31		*n* = 28	
		TB	Non-TB	TB	Non-TB	TB	Non-TB
Characteristics		*n* = 34	*n* = 33	*n* = 20	*n* = 11	*n* = 10	*n* = 18
Female, *n* (%)	56 (44%)	17 (50%)	14 (42%)	7 (35%)	5 (45%)	5 (50%)	8 (44%)
Age, years, median [IQR]	27 [8–41]	27 [7–38]	13 [7–49]	32 [23–50]	20 [6–41]	27 [19–34]	40 [26–50]
Children (< 15 years), *n* (%)	41 (33%)	11 (32%)	18 (55%)	3 (15%)	4 (36%)	2 (20%)	3 (17%)
HIV status, *n* (%)							
HIV positive	20 (16%)	7 (21%)	2 (6%)	5 (25%)	0 (−)	2 (20%)	4 (22%)
HIV negative	74 (59%)	25 (74%)	10 (30%)	15 (75%)	6 (55%)	6 (60%)	12 (67%)
Unknown	32 (25%)	2 (6%)	21 (64%)	0 (−)	5 (45%)	2 (20%)	2 (11%)
Comorbidities, *n* (%)							
Diabetes	4 (3%)	0 (−)	1 (3%)	1 (5%)	1 (9%)	1 (10%)	0 (−)
Heart disease/HT	12 (10%)	2 (6%)	3 (9%)	0 (−)	2 (18%)	1 (10%)	4 (24%)
COPD/Asthma	7 (6%)	3 (9%)	2 (6%)	0 (−)	1 (9%)	1 (10%)	0 (−)
Outpatient, *n* (%)	76 (60%)	32 (94%)	32 (97%)	7 (35%)	0 (−)	3 (30%)	2 (11%)

Abbreviations: *TB* tuberculosis, *IQR* interquartile range, *HIV* human immunodeficiency virus, *HT* hypertension, *COPD* chronic obstructive pulmonary disease

^a^ TB cases: peritonitis (*n* = 6), meningitis (*n* = 2), pericarditis (*n* = 1), spondylitis (*n* = 1); non-TB cases: peritonitis (*n* = 10), meningitis (*n* = 6), mastitis (*n* = 1), osteomyelitis (*n* = 1)

Among the 64 TB cases, 10 (16%) died before first follow-up, and 2 (3%) were lost-to-follow. Thus, 52 TB patients were followed during treatment. Forty patients received standard first-line anti-TB regimen (isoniazid (H), rifampicin (R), pyrazinamide (Z) and ethambutol (E), 2HRZE/4HR), 2 patients an extended standard first-line regimen, 7 children received a regimen without ethambutol (2HRZ/4HR), whereas 3 patients received a retreatment regimen containing first-line drugs including streptomycin (S) (2HRZES/1HRZE/5HRE). An overall clinical improvement was noted in all 52 patients after 2 months of treatment (one patient only assessed after 5 months), but a transient paradoxical reaction with increase in lymphadenopathy was observed in 2 HIV positive patients. Empirical anti-TB treatment was initiated in 12/62 (19%) non-TB patients. Among these, 5/12 patients received a malignant diagnosis, 5/12 cases showed no response to treatment and were further investigated for an alternative diagnosis, 1/12 patients showed clinical improvement before starting anti-TB treatment and 1/12 cases (presumptive TB meningitis) only received anti-TB treatment for 2 weeks due to side-effects and improved without anti-TB therapy.

### TB and non-TB patients present with similar symptoms

Table 2 presents the distribution of symptoms among TB and non-TB cases according to site of infection, age and HIV status. There was no significant difference in the frequency or type of constitutional symptoms when comparing TB with non-TB patients. All patients reported local symptoms from the site of infection. Among the TB cases, patients with lymphadenitis reported less frequently constitutional symptoms as compared to patients with pleuritis and other sites of infection. Paediatric patients described less constitutional symptoms compared to adults. Weight loss, reduced appetite or fatigue were reported more often in adults. Constitutional symptoms were more frequent in HIV positive patients.

**Table 2:** Distribution of constitutional symptoms reported at the time of inclusion, *n* (%)^a^

	Total *n = 126*	Symptoms						
		Constitutional^b^		Fever	Weight loss	Night sweats	Fatigue	Loss of appetite
		≥1 symptoms	≥3 symptoms					
Diagnosis								
TB	64	50 (78%)	23 (36%)	33 (52%)	36 (57%)	21 (33%)	16 (26%)	20 (32%)
Non-TB	62	42 (68%)	22 (35%)	26 (42%)	25 (42%)	18 (29%)	24 (39%)	18 (30%)
*P* value^c^		.189	.958	.279	.086	.604	.109	.741
TB cases								
Confirmed TB	12	9 (75%)	3 (25%)	6 (50%)	6 (50%)	3 (25%)	3 (27%)	4 (33%)
Probable TB	34	28 (82%)	16 (47%)	21 (62%)	23 (70%)	12 (35%)	8 (24%)	13 (38%)
Possible TB	18	13 (72%)	4 (22%)	6 (33%)	7 (39%)	6 (35%)	5 (29%)	3 (19%)
Site								
Lymphadenitis								
TB	34	22 (65%)	7 (21%)	14 (41%)	15 (45%)	7 (21%)	2 (6%)	5 (15%)
Non-TB	33	15 (45%)	6 (18%)	9 (27%)	8 (25%)	4 (12%)	5 (16%)	7 (21%)
Pleuritis								
TB	20	20 (100%)	12 (60%)	13 (65%)	16 (80%)	11 (55%)	9 (47%)	12 (63%)
Non-TB	11	11 (100%)	6 (55%)	7 (64%)	6 (55%)	7 (64%)	8 (73%)	4 (40%)
Other sites								
TB	10	8 (80%)	4 (40%)	6 (60%)	5 (50%)	3 (33%)	5 (50%)	3 (30%)
Non-TB	18	16 (89%)	10 (56%)	10 (56%)	11 (65%)	7 (39%)	11 (61%)	7 (39%)
Age								
Children								
TB	16	12 (75%)	2 (13%)	8 (50%)	5 (31%)	6 (38%)	1 (6%)	0 (−)
Non-TB	25	14 (56%)	6 (24%)	8 (32%)	6 (25%)	6 (24%)	7 (28%)	6 (25%)
Adults								
TB	48	38 (79%)	21 (44%)	25 (52%)	31 (66%)	15 (32%)	15 (33%)	20 (43%)
Non-TB	37	28 (76%)	16 (43%)	18 (49%)	19 (53%)	12 (32%)	17 (47%)	12 (32%)
HIV status^d^								
HIV positive								
TB	14	13 (93%)	6 (43%)	8 (57%)	8 (62%)	5 (38%)	6 (46%)	6 (43%)
Non-TB	6	6 (100%)	4 (67%)	6 (100%)	3 (60%)	1 (17%)	4 (67%)	3 (50%)
HIV negative								
TB	46	33 (72%)	16 (35%)	21 (46%)	27 (59%)	14 (30%)	9 (20%)	14 (32%)
Non-TB	28	20 (71%)	10 (36%)	11 (39%)	15 (54%)	8 (29%)	11 (39%)	8 (29%)

Abbreviations: *TB* tuberculosis, *HIV* human immunodeficiency virus

^a^ Patients with missing values excluded

^b^ The following were included as constitutional symptoms; fever, weight loss, night sweats, fatigue, loss of appetite. ≥1 and ≥ 3 refers to number of patients reporting one or more or three or more symptoms respectively

^c^*P* value < 0. 0071 (0.05/7) considered statistically significant

^d^ Patients with unknown HIV status excluded

### Findings in presumptive TB lymphadenitis

There was no statistically significant difference in the site or symmetry of lymph node involvement between TB and non-TB patients (Table 3). Still, a higher proportion of larger lymph nodes > 4 cm, matted or painful nodes and discharge/fistulas were seen in TB patients compared to non-TB patients.

**Table 3:** Findings in presumptive TB lymphadenitis at the time of inclusion, *n* (%)

		Diagnosis		
	Total	TB	Non-TB	*P* value^a^
Characteristics	*n* = 67	*n* = 34	*n* = 33	
Localization				.549^b^
Unilateral	40 (60%)	22 (65%)	18 (55%)	
Bilateral/generalized	27 (40%)	12 (35%)	15 (45%)	
Only cervical	50 (75%)			
Unilateral		17 (50%)	14 (42%)	
Bilateral		9 (26%)	10 (30%)	
Only axillary	6 (9%)			
Unilateral		3 (9%)	3 (9%)	
Bilateral		0 (−)	0 (−)	
Only inguinal	2 (3%)			
Unilateral		0 (−)	1 (3%)	
Bilateral		0 (−)	1 (3%)	
Cervical and axillary	6 (9%)			
Unilateral		2 (6%)	0 (−)	
Bilateral		1 (3%)	2 (6%)	
Cervical bilateral and axilla unilateral		1 (3%)	0 (−)	
Generalized	3 (4%)	1 (3%)	2 (6%)	
Size of largest lymph node^c^				.031
≤ 2 cm	9 (13%)	1 (3%)	8 (24%)	
> 2–4 cm	25 (37%)	13 (38%)	12 (36%)	
> 4 cm	33 (49%)	20 (59%)	13 (39%)	
Matted nodes	38 (57%)	24 (71%)	14 (42%)	.020
Painful	9 (13%)	7 (21%)	2 (6%)	.081
Discharge/fistula	7 (10%)	6 (18%)	1 (3%)	.051

Abbreviation: *TB* tuberculosis

^a^*P* value < 0. 01 (0.05/5) considered statistically significant

^b^ Test for different risk for unilateral vs. bilateral/generalized localization in TB and Non-TB group

^c^ Long axis of largest lymph node swelling

**Fig. 1:**
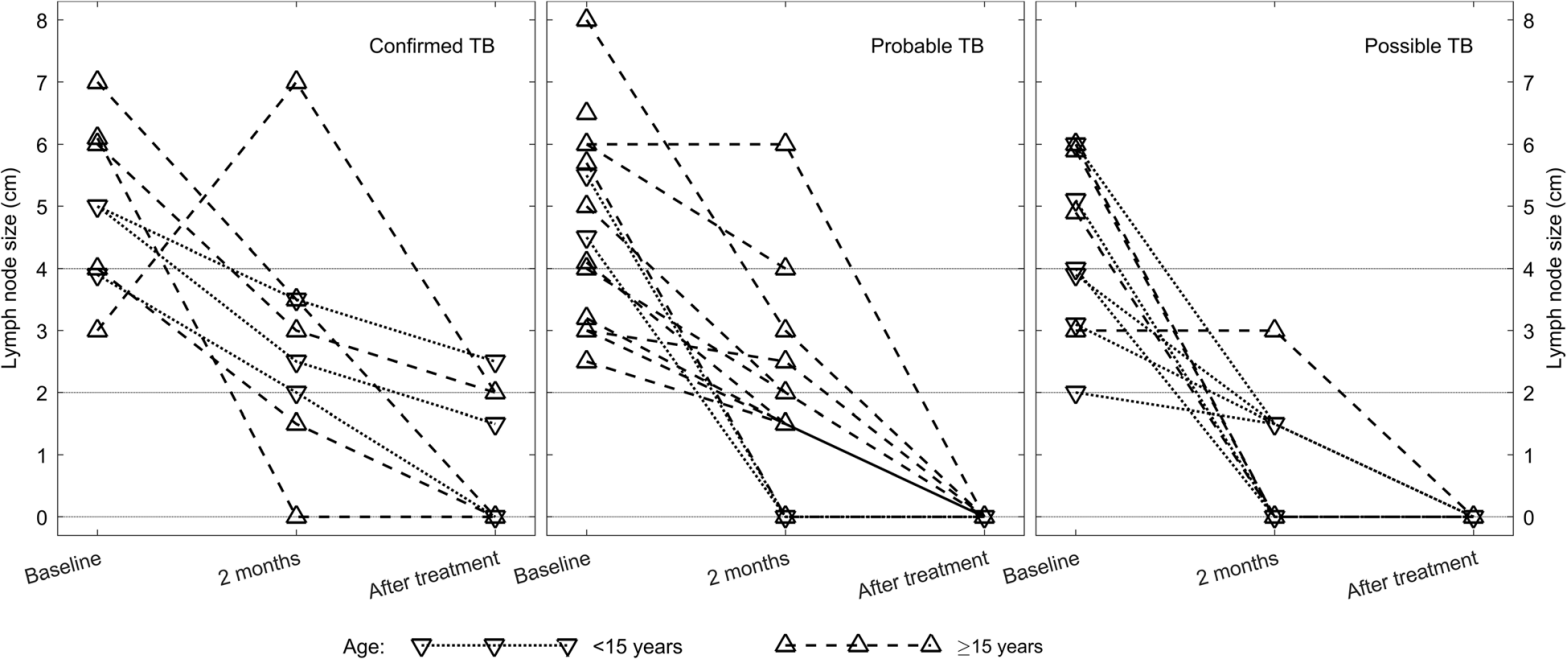
Time series plot illustrating the regression of lymphadenopathy during anti-TB treatment. The lymph node size (long axis of largest swelling) is presented at baseline, after 2 months of treatment and at treatment completion according to TB category (confirmed, probable and possible TB cases) and age. Each patient is represented by an individual line. At baseline, all, except one case, had lymph node enlargement > 2 cm. At 2 months, there was a trend in regression of lymph node swellings, and at treatment completion, 4 (14%) cases had residual lymphadenopathy. The patients with residual lymphadenopathy were all confirmed TB cases. Abbreviation: TB, tuberculosis

There was a significant regression in lymph node size with anti-TB treatment, as shown in Fig. 1. During treatment enlargement of nodes, new fistulae or fresh abscesses were reported in 5 (2 HIV positive) patients. However, overall reduction in lymph node size was seen as an indicator for treatment response among TB patients.

### Regression of pleural and peritoneal effusion during anti-TB treatment

Among the 20 TB pleuritis cases, any regression of effusion was assessed by chest x-ray in 15 patients after 2 months and in 13 patients at treatment completion. Regression was noted in 14 (93%) and 13 (100%), respectively. Only 3/6 (50%) with abdominal TB were evaluated, and partial regression of peritoneal effusion was seen at 2 months and full regression at the end of treatment, as assessed by ultrasound.

### Weight gain as indicator for treatment efficacy

**Fig. 2:**
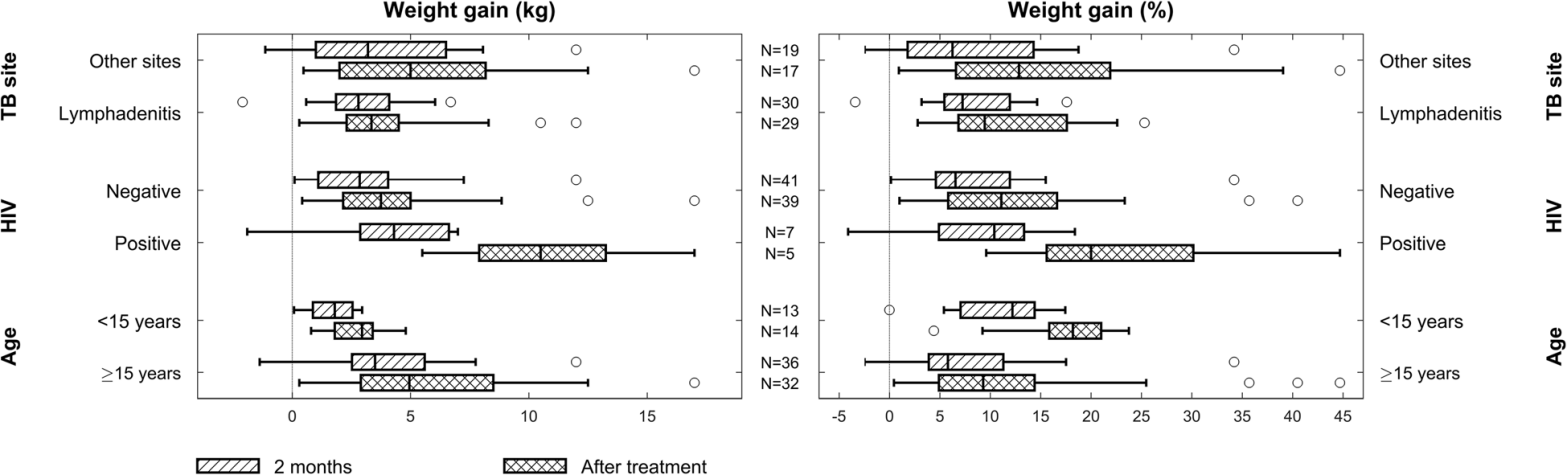
Boxplots showing the weight change at 2 months of anti-TB treatment and at treatment completion. Left boxplot: weight change in kg between treatment onset and 2 months of treatment and between treatment onset and end of treatment according to site of TB infection, HIV status and age. Right boxplot: weight change as percentage change in weight between treatment onset and 2 months of treatment and between treatment onset and end of treatment according to site of TB infection, HIV status and age. Abbreviations: TB, tuberculosis; HIV, human immunodeficiency virus

Weight change during anti-TB treatment was recorded according to age, site of infection and HIV status (Fig. 2). After 2 months of treatment, 36/49 (73%) of the patients had ≥5% weight gain and 18/49 (37%) had ≥10% weight gain. At treatment completion, 38/46 (83%) of the cases had ≥5% and 26/46 (57%) ≥10% weight gain. Overall, the median weight gain at 2 months of treatment was 2.9 kg (interquartile range (IQR), 1.4–5.1 kg) and the median percentage weight gain 6.7% (IQR, 4.6–13.4%). After completion of treatment, the overall median weight gain was 4.0 kg (IQR, 2.2–7.5 kg) and 12.9% (IQR, 7.2–19.7%). The paediatric cases had a higher median percentage weight gain compared to the adult patients both at 2 months (11.1% vs 6.2%) and end of treatment (17.9% vs 9.5%), but the differences were not statistically significant. A weight gain ≥15% was described in 71% of the paediatric TB cases at treatment completion. In all HIV positive cases (*n* = 5) ≥5% weight gain was seen after treatment completion, and 4/5 patients had a weight gain ≥15%. There was no statistically significant difference in weight gain according to site of disease.

### Combination of clinical parameters for assessment of treatment outcome

**Fig. 3:**
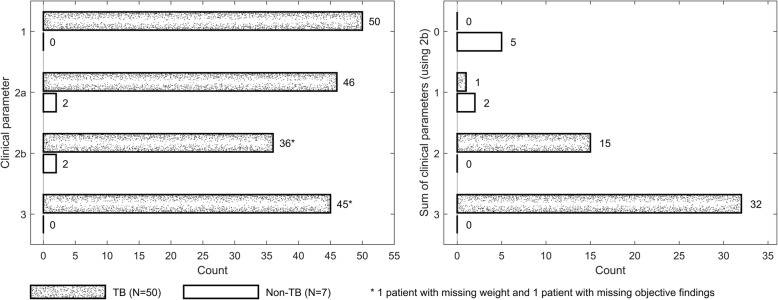
Clinical parameters at 2 months of treatment for assessment of treatment outcome. Left figure: Shows the number of patients having each clinical parameter. 1 – improvement of reported symptoms such as coughing, fever, appetite, fatigue and general condition. 2a – any weight gain compared to baseline; 2b - ≥5% weight gain compared to baseline; 3 - regression of objective findings. Right figure: Shows the number of patients having the sum of clinical parameters. Abbreviation: TB, tuberculosis

Among the patients starting anti-TB treatment, a combination of the following three clinical parameters were assessed retrospectively; 1) Improvement of reported symptoms; 2) Weight gain (any weight gain or ≥ 5% gain); 3) Regression of lymph node swelling or pleural or peritoneal effusion or other local findings, during and after treatment compared to baseline. If a weight gain ≥5% was used, all TB cases, except one, had ≥2 parameters after 2 months of treatment, while non-TB cases fulfilled only 0 or 1 parameter (Fig. 3).

## Discussion

In this study, we describe the clinical presentation of various forms of EPTB and changes in clinical parameters during the course of anti-TB treatment. Our results show that presenting signs and symptoms alone could not differentiate TB and non-TB cases among presumptive EPTB cases. There was regression in clinical signs in TB cases during treatment, and we suggest that a combination of 2–3 clinical parameters could predict good response early (2 months) in the treatment. These findings imply that in low-resource settings where anti-TB treatment is often started on clinical assumption, unnecessary over-treatment can be reduced by systematic clinical monitoring of responses early during treatment.

Improvement in symptoms can be assessed by a symptoms count ratio [3], or self-rated health using the EQ-5D 3 level version [8]. Overall clinical improvement or performance status can be assessed by the health care worker using standardised systems, like the Karnofsky performance score [3, 9]. However, clinically relevant cut-off points for symptoms count ratio, improvement of self-rated health, performance scores and other clinical parameters such as weight gain need to be established in EPTB patients.

Weight gain is an easy, inexpensive parameter for assessing treatment response. Earlier studies, predominantly among PTB patients, have suggested an association with weight gain and successful treatment outcome [10], whereas lesser weight gain or weight loss were associated with poor outcome [10–12] or relapse [13]. Only a few studies have evaluated weight gain as a marker of treatment response in EPTB patients [3, 10, 12]. In our cohort of TB cases with successful treatment outcome, ≥5% weight gain was recorded in 73 and 83% of the cases at 2 months and at treatment completion.

Regression of lymphadenopathy could also be a reliable parameter for assessment of response to treatment, but appearance of newly enlarged nodes, enlargement of existing nodes, formation of new fistulae and abscesses during and after treatment [14, 15] can complicate the evaluation. Paradoxical reactions during anti-TB treatment [16], are described to occur quite frequently in TB/HIV co-infected patients [17, 18]. In a meta-analysis, the frequency was estimated to be 15.7% among patients treated for active TB and newly commencing antiretroviral therapy [16, 19]. Further, paradoxical reactions are reported to be relatively common in HIV negative EPTB patients [20–25]. Therefore, a single clinical parameter may not be enough, and a combination of parameters will be better to differentiate responders from non-responders.

The failure to treatment response could indicate not just alternative diagnoses but also poor compliance, paradoxical reactions or drug resistance in TB cases. These can be difficult to differentiate using only simple clinical parameters. Still, such criteria, incorporated systematically in the follow-up of TB patients, such as in TB registers or treatment cards, can be valuable and assist the health care provider in detecting patients who need clinical reassessment.

Current practice in the TB programme (in Zanzibar) does not detect EPTB patients with poor clinical response. The TB register and treatment cards document treatment completion as the only outcome for EPTB which is based only on the intake of drugs without concurrent record of clinical parameters. The combination of clinical parameters described in this study could be developed into an assessment tool, which can be attached to the patients TB treatment cards. However, the study sample is small, and the findings need to be validated on a larger number of patients. This simple tool has the potential to detect non-responders early at 2 months and standardize follow-ups. Early detection of non-responders would save costs, reduce morbidity due to side-effects, and further minimise the undue delay of alternative diagnoses.

The study has some limitations. Single centre study and the small sample size limits the generalisability of the results and sub-group analysis. There are few patients included with presumptive neurological or bone TB, and none with presumptive genital TB, urinary tract TB or other forms of abdominal TB besides peritoneal TB. Therefore, the results may not apply to all patients presenting with presumptive EPTB in Zanzibar. The possible TB cases were defined by clinically suspected EPTB and overall response to treatment. This does not provide an accurate diagnosis, as some of the patients may have other infections responding to anti-TB treatment. There was also a limited capacity to perform investigations to diagnose other infections and for the HIV patients CD4 counts and viremia were not known. The lack of standardized questionnaire and clinical registration during follow-up and standardized measurements of lymph node size may have influenced the results.

## Conclusions

Clinical presentation alone cannot reliably diagnose EPTB, and empirical anti-TB treatment leads to over-treatment. A combination of simple clinical parameters could be used as an easy tool to assess treatment responses and thus improve patient management in low-resource settings. More and larger studies are needed for further evaluation and validation of these simple clinical parameters.

## Abbreviations

AFB: Acid-fast bacilli
COPD: Chronic obstructive pulmonary disease
CRS: Composite reference standard
E: Ethambutol
EPTB: Extrapulmonary tuberculosis
H: Isoniazid
HIV: Human immunodeficiency virus
HT: Hypertension
IQR: Interquartile range
kg: Kilogram
MMH: Mnazi Mmoja Hospital
Mtb: *Mycobacterium tuberculosis*
PTB: Pulmonary tuberculosis
R: Rifampicin
S: Streptomycin;
TB: Tuberculosis
Xpert: GeneXpert® MTB/RIF
Z: Pyrazinamide
